# Exploring the Effect of Structure-Based Scaffold Hopping on the Inhibition of Coxsackievirus A24v Transduction by Pentavalent N-Acetylneuraminic Acid Conjugates

**DOI:** 10.3390/ijms22168418

**Published:** 2021-08-05

**Authors:** Emil Johansson, Rémi Caraballo, Daniel L. Hurdiss, Nitesh Mistry, C. David Andersson, Rebecca F. Thompson, Neil A. Ranson, Georg Zocher, Thilo Stehle, Niklas Arnberg, Mikael Elofsson

**Affiliations:** 1 Department of Chemistry, Umeå University, SE90187 Umeå, Sweden; emil.johansson@umu.se (E.J.); remi.caraballo@umu.se (R.C.); cd.andersson@gmail.com (C.D.A.); 2 Virology Section, Infectious Diseases and Immunology Division, Department of Biomolecular Health Sciences, Faculty of Veterinary Medicine, Utrecht University, 3584 CL Utrecht, The Netherlands; d.l.hurdiss@uu.nl; 3 Department of Clinical Microbiology, Section of Virology Umeå University, SE90185 Umeå, Sweden; nitesh.mistry@umu.se (N.M.); niklas.arnberg@umu.se (N.A.); 4 Astbury Centre for Structural Molecular Biology, Faculty of Biological Sciences, University of Leeds, Leeds LS2 9JT, UK; r.f.thompson@leeds.ac.uk (R.F.T.); n.a.ranson@leeds.ac.uk (N.A.R.); 5 Interfaculty Institute of Biochemistry, University of Tübingen, 72076 Tübingen, Germany; georg.zocher@uni-tuebingen.de (G.Z.); thilo.stehle@uni-tuebingen.de (T.S.); 6 Department of Pediatrics, Vanderbilt University School of Medicine, Nashville, TN 37232, USA

**Keywords:** sialic acid conjugates, antivirals, conjunctivitis, coxsackievirus A24v, 5-*N*-acetylneuraminic acid, multivalency, cryo-EM

## Abstract

Coxsackievirus A24 variant (CVA24v) is the primary causative agent of the highly contagious eye infection designated acute hemorrhagic conjunctivitis (AHC). It is solely responsible for two pandemics and several recurring outbreaks of the disease over the last decades, thus affecting millions of individuals throughout the world. To date, no antiviral agents or vaccines are available for combating this disease, and treatment is mainly supportive. CVA24v utilizes Neu5Ac-containing glycans as attachment receptors facilitating entry into host cells. We have previously reported that pentavalent Neu5Ac conjugates based on a glucose-scaffold inhibit CVA24v infection of human corneal epithelial cells. In this study, we report on the design and synthesis of scaffold-replaced pentavalent Neu5Ac conjugates and their effect on CVA24v cell transduction and the use of cryogenic electron microscopy (cryo-EM) to study the binding of these multivalent conjugates to CVA24v. The results presented here provide insights into the development of Neu5Ac-based inhibitors of CVA24v and, most significantly, the first application of cryo-EM to study the binding of a multivalent ligand to a lectin.

## 1. Introduction

Viral pandemics have over the course of human civilization, caused the suffering and deaths of millions of human beings [1]. The severe consequences of viral outbreaks and pandemics are currently on global display, with the ravaging covid-19 pandemic that have crippled societies causing serious and, potentially, long-lasting socio-economic effects at both a local level and on a global scale.

Coxsackievirus A24 variant (CVA24v), an antigenic variant of coxsackievirus A24 (CVA24), has over the last decades caused two pandemics and several recurring outbreaks of the highly contagious eye infection acute hemorrhagic conjunctivitis (AHC) with >10 million estimated cases of the disease [2,3]. AHC is characterized by an abrupt onset of ocular pain, swelling of the eyelids, watering, a foreign body sensation, and extensive subconjunctival hemorrhaging [4]. The infection is highly contagious with short incubation periods (24–48 h) that result in explosive outbreaks in communities, affecting up to 50% of the population [3,4,5]. AHC generally resolves spontaneously within 1–2 weeks, and although severe conditions are rare, the development of acute flaccid paralysis and even fatalities have been reported [5,6]. Treatment of AHC is supportive as neither vaccines nor antivirals are currently available to combat the infection.

To facilitate attachment and subsequent entry into cells, CVA24v engages glycan-containing receptors that terminate in 5-*N*-acetyl-neuraminic acid (Neu5Ac) and the intercellular adhesion molecule-1 (ICAM-1), with ICAM-1 serving as the primary receptor [7]. Binding to ICAM-1 is essential for productive replication of CVA24, including variant and non-variant strains, as binding initiates uncoating by triggering destabilizing rearrangements of the capsid proteins that eventually result in the formation of a capsid-mediated pore through which the viral genome is released into the cytoplasm of cells [7]. However, the virulence and pandemic potential of CVA24v seems to be linked to enhanced efficiency in binding Neu5Ac, which favors a tropism switch towards the eye [7]. This adaptation has further been suggested to promote CVA24v attachment to corneal and conjunctival cells [8] and thereby, resulting in enhancing the transmission of viral progeny [7]. CVA24v binds Neu5Ac glycans via binding sites located within viral protein 1 (VP1) that together cluster around the five-fold symmetry axis of the virion, in essence, forming pentameric Neu5Ac lectins [9].

Protein-carbohydrate interactions are of vital importance in a wide range of biological processes in health and disease. Yet, only a few carbohydrate-based drugs have reached the market. This may be due to challenges associated with overcoming the generally weak affinities of protein–carbohydrate interactions and the poor pharmacological properties of carbohydrates as a result of their high polarity and metabolic vulnerability [10]. In the case of CVA24v and many other viruses that employ Neu5Ac for host cell attachment, these issues can potentially be circumvented by design of multivalent ligands intended for a topical administration as the primary sites of replication are linked to the eyes and airways. Importantly, direct topical administration to the site of infection would circumvent the challenges posed by oral administration and systemic distribution of a carbohydrate drug. Indeed, the design of multivalent carbohydrate ligands is a proven strategy used to achieve high avidity binding to lectins (carbohydrate-binding proteins) via several mechanisms, including the chelate effect, subsite binding, steric stabilization, statistical rebinding, and receptor clustering [11]. Further, multivalent ligands tailored to lectin valency and topology have been demonstrated to result in rather exceptional enhancements in avidity [12,13]. Other promising approaches include repurposing existing topical eye medications to be used as broad-spectrum antivirals [14].

Bioisosteric replacements are broadly defined as the replacement of, e.g., functional groups or a scaffold part of a bioactive molecule with a substructure that is similar in size and properties. The strategy is widely used in compound optimization with the aim of improving target binding, drug-like properties, reduce metabolic vulnerability, or reduce toxicity. Replacement of the central scaffold of an active molecule with another scaffold has in recent years gained increased attention [15]. This is the result of, e.g., development of computational methods referred to as “scaffold hopping” [16] and the realization that the diversity of scaffolds in approved drugs are relatively low [17]. Whether the selection of a novel scaffold for an active compound is based on “scaffold hopping” methodology or on traditional structure-based reasoning [18], the general idea is to retain enough similarity for the compound to remain active, normally achieved by keeping key substituents for binding. This may result in altering the properties of the molecule or may move it into novel chemical space [15,16]. Scaffold-replacement has been demonstrated to result in improved binding affinity [19] and cell permeability [20], and reduced toxicity [21]. Further, decreased flexibility of the scaffold can increase the binding affinity of a molecule as the loss of conformational entropy is reduced upon binding of the ligand to its target [22].

To date, pentavalent Neu5Ac conjugates based on a glucose scaffold are the most efficient reported inhibitors of CVA24v attachment and infection of human corneal epithelial (HCE) cells [23]. Herein, we report on the synthesis of scaffold-replaced pentavalent Neu5Ac conjugates and, to the best of our knowledge, the first use of cryogenic electron microscopy (cryo-EM) to study the dynamic nature of a multivalent ligand binding to CVA24v.

## 2. Results

### 2.1. Design

A total of 60 Neu5Ac binding sites decorate the CVA24v capsid. These are arranged into 12 relatively flat, local five-fold symmetries, each providing five shallow, surface exposed Neu5Ac binding sites [9]. We previously reported on the design and synthesis of pentavalent Neu5Ac conjugates with pseudoradial topology that inhibit CVA24v attachment to, and infection of, HCE cells [23]. These inhibitors were designed to chelate each of the pentameric lectins of CVA24v. Crystal structures of inhibitor-bound CVA24v confirmed compound binding, with observed electron density for the Neu5Ac units. However, negative-staining electron microscopy suggested that the major mode of binding and inhibition of these inhibitors likely resulted from cross-linking and aggregation of virus particles [23]. Thus, in order to further explore, and achieve, tailored chelation of the Neu5Ac binding sites, a modification of the strategy was needed.

We reasoned that replacement of the glucose scaffold of the pentavalent Neu5Ac conjugates potentially could result in direct contacts between the scaffold and the viral capsid and thus result in improved efficacy. We hypothesized that tailored pentavalent Neu5Ac conjugates with symmetric topology may facilitate detection of the remaining parts of the inhibitors as the number of unique binding modes are significantly reduced, and could thus result in more ordered binding events to the viral capsid. Theoretically, this should result in higher avidity binding due to reduced loss of entropy in the inhibitor-lectin interaction [24,25,26]. Further, although many different multivalent scaffold architectures have been developed, the effect of scaffold-replacements in the optimization of multivalent ligand structures is a rather underexplored topic as most reports have focused on ligand valency and density and on the refinement of spacer length and characteristics [24].

Penta-butynyl-corannulene [27] was selected as the scaffold based on its five-fold symmetry, equivalent to that of CVA24v, and due to the synthetic ease of accessing C5-symmetric pentavalent Neu5Ac conjugates via the copper-catalyzed azide-alkyne cycloadditions (CuAAC). The scaffold may interact with amino acid residues of the capsid via CH-π, cation-π, or π-π interactions [28,29]. In addition, the aromatic bowl-shaped backbone of the corannulene scaffold imparts a dipole moment, through which interactions could be induced [28]. Furthermore, bowl-inversions of the scaffold occur readily [30,31], thus, either the concave or convex face of the scaffold can interact with the virus particle, which may be ideal due to the spherical form of CVA24v.

**Figure 1:**
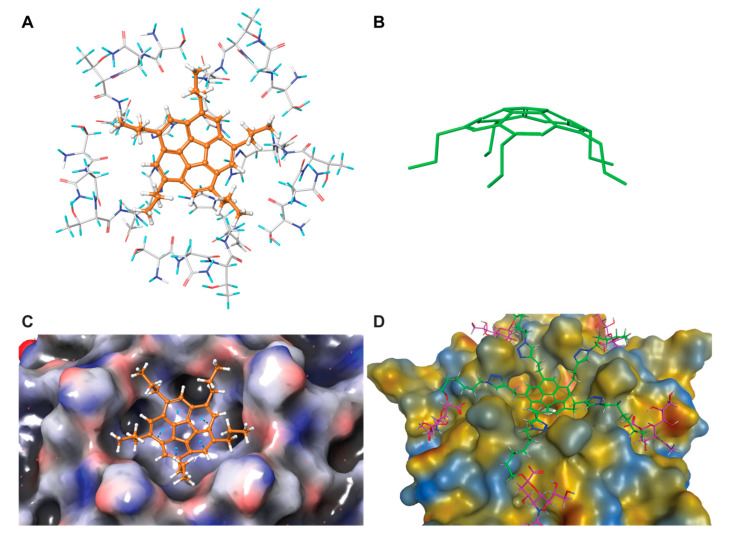
Energy minimization and modelling of scaffold and a pentavalent Neu5Ac conjugates to CVA24v. (**A**) penta-propyl-corannulene and CVA24v atoms were included in the docking. (**B**) Energy minimized structure of the penta-propyl-corannulene. (**C**) Quantum mechanical molecular modeling of penta-propyl-corannulene scaffold to a truncated part of the “lectin receptor” of CVA24v. (**D**) Molecular mechanical modeling of a scaffold replaced corannulene-based pentavalent Neu5Ac conjugate to CVA24v.

Quantum mechanical molecular modeling was used to probe potential interactions between the corannulene-scaffold and CVA24v. To reduce the computational demands of the quantum mechanical calculations, a penta-propyl substituted corannulene molecule was docked to a truncated region, consisting of 340 atoms, of one pentagon of CVA24v that included the five-fold symmetry axis of the virion (Figure 1A).

The docking started with a low-energy penta-propyl-substituted corannulene (Figure 1B) that was manually placed on top of the five-fold center of CVA24v. Subsequently, the complex was geometry optimized in Jaguar [32] using density functional theory (BLYP-D3 [33], 6-31G **), with the molecule allowed to move freely over the viral surface with fixed atomic positions. The optimization converged after 48 cycles according to the direct inversion of the iterative space method [34]. No atomic clashes were identified, and beneficial arene interactions were present between histidine side chains and the corannulene molecule (Figure 1C). This docked structure was then used to study the length of the spacers required for chelation of the pentameric lectins (Figure 1D) [23]. The docked structure of corannulene was connected to the sialic acids (X-ray coordinates), and the spacers were energy minimized. Spacers of increasing length were evaluated to minimize the strain where *n* = 1 led to a strained spacer and *n* = 5 was sufficiently long to avoid strain.

### 2.2. Synthesis of Scaffold-Replaced Pentavalent Neu5Ac Conjugates

**Scheme 1:**
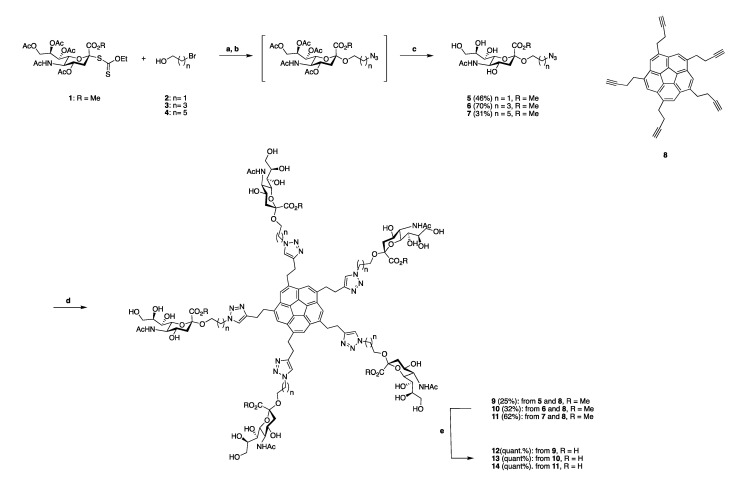
Synthesis of pentavalent corannulene-based Neu5Ac compounds. Reagents and conditions: (**a**) i: molecular sieves (3 Å), **2**−**4**, CH_3_CN/CH_2_Cl_2_ (2:1), rt, 30 min, ii: silver trifluoromethane-sulfonate, IBr, −74 °C, 5.5 h. (**b**) NaN_3_, DMF, (**c**) Sodium methoxide, methanol, rt, 3 h, ii: H^+^ ion exchange resin. (**d**) CuSO_4_·5H_2_O, sodium ascorbate, tetrahydrofuran/H_2_O (1:1), **8**, 50 °C, 5 h → rt, 18 h. (**e**) i: LiOH, methanol, rt, 24 h, ii: H^+^ ion exchange resin.

Synthesis of the symmetric pentavalent Neu5Ac conjugates **12**–**14** was achieved in five steps from Neu5Ac donor **1** that was prepared from Neu5Ac as previously described (Scheme 1) [35].

The sym-penta-(1-butyn-4-yl)-corannulene core **8** [27] was obtained in three steps from corannulene under standard conditions [27,31]. Neu5Ac donor **1** was sialidated using the glycosyl acceptors **2**–**4**, respectively, under the promotion of silver trifluoromethanesulfonate and iodine monobromide [36], affording the corresponding bromo acetyl protected sialosides. Subsequent treatment with sodium azide in dimethylformamide (DMF), followed by *O*-deacetylation afforded the known α-anomerically pure azido sialosides **5** [13] and **6** [23], in 46% and in 70% yields, respectively, while **7** was isolated in 31% yield over the three steps. The azido sialosides **5**–**7** were then conjugated to the pentapropargylated core **8** by the copper-catalyzed azide-alkyne cycloaddition affording the pentavalent Neu5Ac methyl esters **9**–**11** in 25–62% yields. Treatment with lithium hydroxide in methanol and water liberated the corresponding pentavalent Neu5Ac compounds **12**–**14** in quantitative yields after neutralization.

### 2.3. Corannulene-Based Pentavalent Neu5Ac Conjugates Attenuate CVA24v Transduction

**Figure 2:**
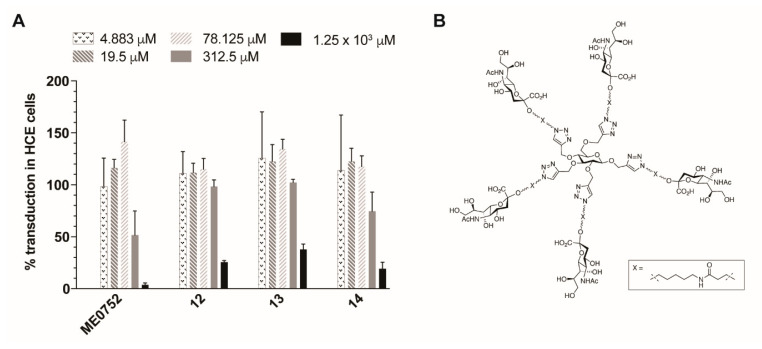
Effect of scaffold replacement on CVA24v transduction in HCE cells. (**A**) Effect of scaffold-replaced pentavalent Neu5Ac conjugates **12–14** on CVA24v transduction in HCE cells at 37 °C. Error bars are shown as the standard error of the mean (SEM). Data are presented as the % of control, that is, the value obtained in the absence of an inhibitor. All experiments were performed in duplicate and for a minimum of two times. (**B**) Structure of glucose-based pentavalent Neu5Ac conjugates **ME0752**.

To evaluate the biological effect of the scaffold-replaced Neu5Ac conjugates **12**–**14**, the transduction of CVA24v at 37 °C was measured in HCE cells (Figure 2A), as previously described [23]. Ocular inspection of the cells by microscopy and cell counting (Figure S1) indicated that the compounds are non-toxic.

Compared with untreated virions, the transduction of CVA24v was significantly attenuated when preincubated with **12**–**14**. All inhibitors were significantly more potent than Neu5Ac [23] and disialyllacto-*N*-tetraose [9]. Control compound **ME0752** with a β-glucose scaffold (Figure 1B, compound **28** in reference [23]), attenuated transduction with >95% and 50% at 1.25 mM and 312.5 μM, respectively, but had no effect at lower concentrations. Compound **14**, with the longest spacers, was the most efficient of the scaffold-replaced compounds and reduced transduction by 80–85% and 25% at 1.25 mM and 312.5 μM, respectively. Compounds **13** and **12**, containing successively shorter spacers, reduced transduction by 75% and 60%, respectively, but had no effect at 312.5 μM. A plausible explanation for this could be that the spacers of compounds **12** and **13** are not of sufficient length to allow simultaneous binding of the Neu5Ac units of the compounds to the pentameric lectins of CVA24v. It is also possible that the compounds cause cross-linking and aggregation as previously observed for glucose-based pentameric sialic acids, including **ME0752** [23]. Nevertheless, the displayed inhibitory activity of compound **14** indicates that the glucose scaffold can be replaced with a corannulene scaffold, although for this particular system and set of compounds, it did not result in enhancing potency.

### 2.4. X-ray Crystallography Supports Binding of Inhibitors to CVA24v

**Figure 3:**
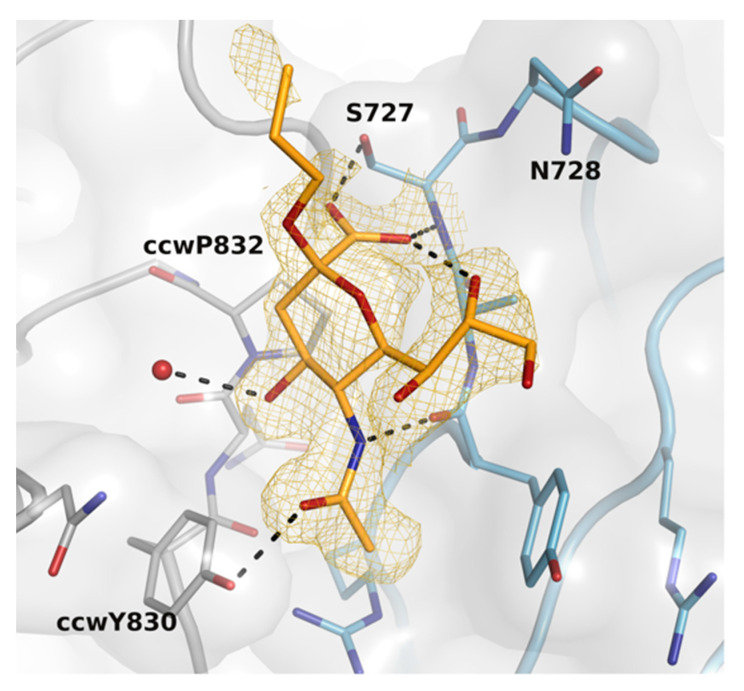
Binding of compound **14** to CVA24v. Interactions of the Neu5Ac moiety of compound **14** to amino acids in the ligand binding sites of CVA24v. The binding site located at the interface of two lectins proteins (light blue, light grey) is occupied by the sialic acid entity (orange) of the inhibitor as shown by an unbiased simulated annealing (Fo-Fc)-map (light orange) depicted at a contour level of 2.5 σ. The spacer to the corannulene core is flexible, resulting in a fuzzy electron density. Therefore, neither the corannulene nor most parts of the spacer can be modeled into the crystal structure.

The binding mode of **14** was structurally characterized by X-ray crystallography. The X-ray structure of **14**-CVA24v was established to a resolution of 1.78 Å. Similar to the CVA24v structures with the glucose-based Neu5Ac inhibitors [23] (pdb code 6TSD), the **14**-CVA24v structure showed clear electron density for the Neu5Ac units in all five binding sites, each located within viral protein 1 (VP1). The electron density of the spacer points outward to the solvent and shows that the spacer does not have specific contacts to the viral shell. This results in a high degree of flexibility in that region, resulting in a blurry map that does not allow the placement of more than the first three atoms of the spacers of compound **14**. Thus, the remaining parts of **14** were not resolved, suggesting that it is either not ordered or that the inhibitor does not accommodate a single conformation. Further, it is possible that **14** does not bind in a chelating fashion similar to the glucose-based pentavalent Neu5Ac conjugates that were observed to cross-link and aggregate CVA24v particles [23]. Thus, **14** may bind to Neu5Ac binding sites of separate CVA24v particles within the crystal lattice, resulting in the absence of observed density as the spacers likely adopt random orientations in the solvent. The interactions in the lectin binding sites of CVA24v with the Neu5Ac units of compound **14** were similar as previously described [23] (Figure 3), with the main exception that the C8 hydroxyl rather than the C9 hydroxyl of Neu5Ac engages in intramolecular hydrogen bonding with the C2 carboxylate.

### 2.5. Cryo-EM Supports Inhibitor Binding over the Five-Fold Axis of CVA24v

**Figure 4:**
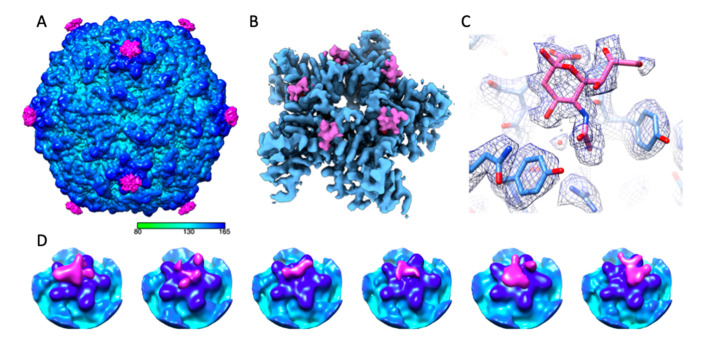
Cryo-EM analysis of CVA24v in complex with compound **14**. (**A**) Unsharpened cryo-EM reconstruction of CVA24v in complex with compound **14**, viewed down the icosahedral two-fold axis. The capsid is colored according to the radial coloring scheme, shown in angstroms, and the density corresponding to compound **14**, visible at the five-fold symmetry axis, is colored magenta. (**B**) Enlarged view of a single capsid five-fold from the sharpened cryo-EM map, showing clear density for the sialic acid component of compound **14**. (**C**) Zoomed in view of a single sialic acid binding site shown in (**B**). (**D**) 3D classes obtained from no-alignments, focused classification of the five-fold symmetry axis, colored as in (**A**).

To understand how compound **14** binds to CVA24v in solution, cryo-EM analysis was performed. To exclude the possibility of cross-linkage between the inhibitor and binding sites of neighboring capsids, we employed an on-grid binding strategy described previously [37,38]. This approach allowed us to determine the **14**-CVA24v structure at an overall resolution of 2.3 Å (Figure 4A).

In the unsharpened map, which retains lower resolution features, a clear mushroom-shaped density for compound **14** was visible at the capsid vertices (Figure 4A). However, upon map sharpening, only the sialic acid component of the inhibitor was resolved at high resolution, with a binding mode virtually identical to that observed in the X-ray crystallography structure (Figure 3 and Figure 4B,C). Because the core and spacer regions of compound **14** were poorly resolved, focused classification [39] was performed on a single five-fold symmetry axis of the CVA24v capsid to study alternative conformations of the inhibitor. In this approach, 60 symmetrically redundant orientations were assigned to each particle, and a mask was used to focus classification on a substructure within the virion. In the absence of symmetry averaging, this approach revealed a striking heterogeneity in the binding mode of compound **14** and demonstrated that the inhibitor density deviates from five-fold symmetry (Figure 4D and Video S1). Moreover, in several classes, compound **14** is only localized to a subset of available sialic acid binding sites. This suggests that only a couple of Neu5Ac appendages of the compound are in contact with the capsid at any one time, and the others adopt random orientations in the solvent. Clearly, this indicates that **14** is not capable of fully chelating the Neu5Ac binding sites of the CVA24v lectins. Thus, it is plausible that a radial presentation of Neu5Ac ligands is not an appropriate design strategy for this particular system, possibly due to the overall topology of the lectin-receptor. The rising of the protein surface from the center of the five-fold symmetry axis of the virion in relation to the Neu5Ac binding sites gives the lectin-receptor an overall convex-concave-convex surface going from one Neu5Ac binding site across the center to another. Computational work studying the stability of complexes between divalent ligands and bivalent receptors with concave (i.e., depression or channel) or convex (i.e., rising or hill) surfaces between the binding sites, have shown that spacer-receptor interactions are particularly detrimental in convex receptors and result in vanishing avidity enhancements [40]. It is possible that similar factors play a role in this particular system as well.

## 3. Conclusions

In this study, we performed structure-based scaffold hopping on pentavalent Neu5Ac conjugates in an attempt to achieve a chelating binding to CVA24v, and evaluated the effect of the scaffold replaced compounds on CVA24v transduction. The rationale for the replacement of a glucose scaffold with corannulene was confirmed by quantum mechanical docking of the corannulene scaffold to CVA24v that indicated the presence of beneficial arene interactions to the protein surface of CVA24v. The effect of the scaffold-replaced pentavalent Neu5Ac compounds generated no beneficial effect on the inhibition of CVA24v transduction in relation to the glucose-based inhibitor **ME0752**. We used cryo-EM to study the binding of the corannulene-based compound **14** to CVA24v, which indicated that binding does not occur via the intended chelation of the binding sites. Thus, we find it reasonable to speculate that this is a major contributing factor to the small observed avidity gains achieved by both the glucose- and corannulene-based pentavalent Neu5Ac conjugates. Likely, a mixture of multivalent binding mechanisms are operating at any given time resulting in the inhibition of CVA24v transduction. This is valuable information for the future development of Neu5Ac-based inhibitors of CVA24v. Future studies could explore the effect of multivalent Neu5Ac conjugates with different topologies.

## 4. Materials and Methods

### 4.1. Computational Modelling

The molecular structure of the penta-propyl-corannulene was energy minimized using the semi-empirical method Austin model 1 (AM1) [41,42] followed by geometry optimization with density functional theory (DFT) in Jaguar (Schrödinger, New York, NY, USA) [32,43] using functional B3LYP [33,43,44] and basis set CC-PVTZ. This resulted in a low-energy structure with the expected bowl-shaped corannulene moiety (Figure 1A). The docking of penta-propyl-corannulene to a truncated region of CVA24v, consisting of 340 atoms of 1 pentagon, started with the manual placement followed by a optimization of the interaction using DFT (BLYP-D3 [33,44,45], 6-31G **) in Jaguar [32,43]. During the geometry optimization, the penta-propyl-corannulene was allowed to move but the virus binding site, including protein atom in close proximity (Figure 1A), had fixed atomic positions. The optimization converged after 48 cycles according to the direct inversion of the iterative subspace method [34]. No atomic clashes were identified, and there was an indication for a beneficial arene interaction between corannulene and the proximal histidine side chains in the binding site (Figure 1B). Using this docked structure, spacers of different lengths to the sialic acid moiety were hypothesized and tested.

### 4.2. CVA24v Production and CVA24v Transduction

CVA24v of high purity (Figure S2) was produced as described previously [8,9] and the CVA24v transduction assay was carried out according to established protocols [23].

### 4.3. Crystallization and Structure Determination

CVA24v was crystallized and derivatized as previously reported [9]. Briefly, compound **14** (17 mM) was soaked overnight at 4 °C before the crystals were harvested and flash-frozen in liquid nitrogen. Data were collected at beamline I03 at Diamond Light Source, Oxford, United Kingdom (UK). Data reduction was performed using XDS [46], followed by molecular replacement using rigid body refinement as implemented in REFMAC5 [47]. Simulated annealing using Phenix [48] was applied to remove model bias. The final model resulted from cyclic real-space refinement using COOT [49] and reciprocal space refinement as implemented in REFMAC5. Strict NCS parameterization was applied in all later refinement steps (Table 1). The structure was validated using MOLPROBITY [50] before structure factor amplitudes and coordinates were deposited to the protein data bank (pdb entry 7OJ7). Figures were generated using PYMOL [51].

### 4.4. Cryo-Electron Microscopy

Cryo-EM grids were prepared by applying 3 μL of purified CV-A24v (9.6 mg/mL) to 400 mesh lacey grids coated in a 3 nm carbon film (Agar Scientific, Essex, UK). The sample was left to adsorb for 30 s, and then the excess sample was removed by manual blotting. On-grid binding of pentavalent Neu5Ac conjugate was then performed by applying 3 μL of a 5 mM solution of compound **14** in PBS to the pre-blotted, CV-A24v-coated grid, and leaving for 30 s before blotting and freezing using a Leica EM GP plunge freeze device (Leica Microsystems, Wetzlar, Germany). The Leica EM chamber temperature was set to 8 °C with 80% relative humidity and liquid ethane was used for sample vitrification. Grids were glow discharged at 20 mA for 30 s prior to application of the sample, using a PELCO easiGlow™ (Ted Pella, Redding, CA, USA). All data sets were collected on a Titan Krios (Thermo Fisher Scientific, Waltham, MA, USA) transmission electron microscope operating at 300 kV, at a nominal magnification of 165,000×. A total of 1443 exposures were recorded using EPU automated acquisition software on a Gatan K2 summit camera operating in super-resolution mode, using a total electron dose of 48.9 e−/Å^2^. Each movie had a total exposure of 10 s and contained 50 fractions. The final calibrated physical pixel size was 0.82 Å. Detailed information on data collection was shown in Table 2.

#### Image Processing

Following cryo-EM data collection, the RELION-3.0 pipeline [52] as used for image processing. Drift correction was first performed on micrograph stacks using MOTIONCOR2 [53], and the contrast transfer function for each was estimated using Gctf [54]. A subset of virus particles was picked manually and subjected to 2D classification, with the resultant classes used as templates for automatic particle picking [55]. Particles were classified through multiple rounds of reference-free 2D classification, and particles in poor quality classes were removed after each round. Using the ‘molmap’ command in UCSF chimera [56], the CV-A24v crystal structure (PDB ID: 4Q4V [9]) was used to generate a 40 Å resolution starting model to use as a reference for 3D autorefinement, with icosahedral symmetry imposed. This reconstruction was postprocessed to mask and correct for the B-factor of the map before taking particles forward to contrast transfer function (CTF) refinement and Bayesian polishing [57]. The final reconstruction at 2.3 Å was computed after correcting for the curvature of the Ewald sphere [52]. The nominal resolution the map was determined according to the ‘gold standard’ Fourier shell correlation (FSC) criterion (FSC = 0.143) [55] and a negative B-factor of −49 Å^2^ was applied during the final post-processing step. The local resolution estimation tool in RELION was used to generate a map filtered by local resolution.

To investigate the conformational heterogeneity of compound **14**, a focused 3D classification approach was employed (as described previously [39]). Briefly, each particle contributing to the final icosahedral-symmetry–imposed reconstruction was assigned 60 orientations corresponding to its icosahedrally related views using the relion_symmetry_expand tool. SPIDER [58] was used to generate a spherical mask, which was manually placed over the map to isolate the fivefold symmetry axis of the capsid, and the symmetry-expanded particles were subjected to masked 3D classification without alignment using a regularization parameter (‘T’ number) of 20. During the no-alignments 3D classification, the resolution in the E-step was limited to 12 Å. Figures were generated using UCSF Chimera [56] and UCSF ChimeraX [59].

### 4.5. General Chemical Procedures

^1^H NMR and ^13^C NMR spectra were recorded with a Bruker DRX-400 spectrometer (Bruker, Billerica, Massachusetts, USA) at 400 MHz and 100 MHz, respectively, or with a Bruker DRX-600 spectrometer at 600 MHz and 150 MHz, respectively. NMR experiments were conducted at 298 K in D_2_O (residual solvent peak = 4.79 ppm (δH), CD_3_OD (residual solvent peak = 3.31 ppm (δH) and 49.00 ppm (δC)), or CDCl_3_ (residual solvent peak = 7.26 ppm (δH) and 77.16 ppm (δC)). Liquid chromatography mass spectrometry (LCMS) were recorded by detecting positive/negative ion (electrospray ionization, ESI) on Agilent 1290 infinity II−6130 Quadrupole (Agilent, Santa Clara, CA, USA) using H_2_O/CH_3_CN (0.1% formic acid) as the eluent system or on Agilent 1290 infinity−6150 Quadrupole using YMC Triart C18 1.9 μm, 20 × 50 mm column (Genetec, Nacka, Sweden) and H_2_O/CH_3_CN (0.1% formic acid) as the eluent system. High resolution mass spectra (HRMS) data were recorded with Agilent 1290 binary LC System connected to a Agilent 6230 Accurate-Mass Time-of-Flight (TOF) LC/MS (ESI+) (Agilent, Santa Clara, CA, USA); calibrated with Agilent G1969-85001 ES-TOF Reference Mix containing ammonium trifluoroacetate, purine and hexakis(1H, 1H, 3H tetrafluoropropoxy)phosphazine in 90:10 CH_3_CN/H_2_O. Semi-preparative HPLC separations were performed on a Gilson system high performance liquid chromatography (HPLC), using a YMC-Actus Triart C18, 12 nm, S-5 μm, 250 × 20 mm (Genetec, Nacka, Sweden) with a flow rate 20 mL min^−1^, detection at 214 nm and eluent system A: aqueous 0.005% formic acid, and B: CH_3_CN 0.005% formic acid. Column chromatography was performed on silica gel (60 Å, 70–230 mesh ASTM). Thin layer chromatography (TLC) were performed on Silica gel 60 F254 (Merck KGaA, Darmstadt, Germany) with detection under UV light and/or development with 5% H_2_SO_4_ in EtOH and heat. Automated flash column chromatography was performed using a Biotage Isolera One system (Biotage Sweden, Uppsala, Sweden) and purchased pre-packed silica gel cartridges (Biotage SNAP Cartridge, KP-Sil). Freeze drying was performed by freezing the diluted CH_3_CN/water solutions in dry ice-acetone bath and then employing a Scanvac CoolSafe freeze dryer connected to an Edwards 28 rotary vane oil pump (Labogene, Allerœd, Denmark). Organic solvents were dried using a Glass Contour Solvent Systems (SG Water, NH, USA) except CH_3_CN (freshly distilled from CaH_2_) and MeOH that were dried over molecular sieves 3 Å. Corannulene and 4-bromo-1-butanol were purchased from abcr GmbH Germany (Karlsruhe, Germany), and all other chemicals from Sigma-Aldrich. All chemicals and were used as received. All target compounds were ≥95% pure according to HPLC UV-traces. Statistics were calculated using GraphPad Prism 7 (GraphPad Software, La Jolla, CA, USA). Microwave reactions were performed using a Biotage Initiator microwave synthesizer (Biotage Sweden, Uppsala, Sweden); temperatures were monitored by an internal IR probe; stirring was mediated magnetically, and the reaction was carried out in sealed vessels. Automated flash column chromatography was performed using a Biotage^®^ Isolera One system and purchased pre-packed silica gel cartridges (Biotage SNAP Cartridge, KP-Sil, Biotage Sweden, Uppsala, Sweden).

#### 4.5.1. General Procedure for Synthesis of Deprotected Azido-Sialosides

An oven-dried round bottom flask was charged with magnetic stirring bar, powdered activated molecular sieves (4Å, 3.3 g), xanthate sialoside donor (1.0913 mmol, 1.0 eq) and placed under N_2_ atmosphere. To this mixture was added CH_2_Cl_2_ (50 mL), bromoalcohol (2, 3, or 4, respectively, 1.20 eq), and under dark conditions, a solution of silver trifluormethansulfonate (2.0 eq) in freshly distilled CH_3_CN (77 mL). The solution was cooled to −72 °C and stirred for 15 min, followed by dropwise addition of a solution of IBr (1.0 M in CH_2_Cl_2_, 1.40 eq). After complete addition of the IBr solution, the reaction was stirred for 2 h at −72 °C, and *N,N*-diisopropylethylamine (DIPEA) (6.0 eq) was added and stirred for another 20 min at −72 °C, and another 20 min before warming to rt. The mixture was filtered through a plug of celite and concentrated to dryness. The resulting mixture was purified by flash chromatography (CH_2_Cl_2_/CH_3_OH gradients), affording the protected sialosides as a mixture of alpha and beta anomers, in addition to the elimination product. The mixture (544 mg, 0.8312 mmol) was subsequently dissolved in DMF (25 mL) and treated with NaN_3_ (6.0 eq) followed by tetra-n-butylammonium iodide (2.0 eq). The reaction was allowed to stir under nitrogen atmosphere for 22 h. The mixture was diluted in CH_2_Cl_2_, washed with water, HCl (1 M), and brine. The organic layer was dried of anhydrous Na_2_SO_4_, filtered and concentrated under reduced pressure affording crude product, which was used without additional purification. The crude was dissolved in a 0.03 M solution of sodium methoxide (a total of 10.0 equiv.). The reaction was allowed to stir at rt under nitrogen atmosphere for 24 h. The solution was neutralized by the addition of Dowex 50 × 8 H-form (pre-washed with MeOH), filtered, and concentrated to dryness. Mixture was purified using column chromatography (CH_2_Cl_2_/MeOH, from 6% MeOH to 10% MeOH), affording the deprotected azido sialosides **5**–**7** as pure α-anomers. See below for specific yields and analytical data.

#### 4.5.2. General Procedure for CuAAC

An oven-dried round bottom flask equipped with magnetic stirring bar was charged with azido-sialoside (0.37 mmol, 11.5 eq). To this was added a solution of pentapropargylated corannulene core 8 (1.0 eq, 0.032 mmol) in tetrahydrofuran (THF, 7 mL). To the stirring solution was added CuSO_4_^.^5H_2_O (1.59 eq) and sodium ascorbate (1.55 eq) in H_2_O (7 mL). The flask was equipped with rubber septa and the mixture was heated to 50 °C for 5 h and then the reaction was left to perform at room temp for 36 h. The THF was removed under reduced pressure, and the resulting mixture was injected on HPLC (MeCN/H_2_O 10% → 25% gradient in 25 min), affording the pentavalent methyl ester derivative after freeze-drying. See chemical synthesis for specific yields and analytical data.

#### 4.5.3. General Procedure for Ester Hydrolysis

The pentavalent methyl ester derivate (0.01 mmol, 1.0 eq) was dissolved in CH_3_OH (1.35 mL), and to this stirring solution was added a 1M solution of LiOH (0.156 mL, 15.0 eq). The mixture was stirred for 48 h at room temperature in the dark. The mixture was neutralized (pH 7–8) with Dowex 50x8 H-form, filtered, and concentrated under reduced pressure. The resulting residue was diluted in water and freeze-dried to afford the pentavalent target compound.

Methyl (2-(6-azidohexoxy)(5-*N*-acetamido-3,5-dideoxy-D-glycero-α-D-galacto-2-nonylopyranosyl))-onate (**7**). ^1^H NMR (600 MHz, CD_3_OD): δ 1.32–1.43 (m, 4H), 1.50–1.52 (m, 4H), 1.73 (dd, *J* = 12.8 Hz, 11.9 Hz, 1H), 2.00 (s, 3H), 2.68 (dd, *J* = 12.9 Hz, 4.7 Hz, 1H), 3.28 (appear as t, *J* = 6.9 Hz, 2H), 3.34–3.39 (m, 1H), 3.51 (dd, *J* = 8.9 Hz, 1.8 Hz, 1H), 3.55 (dd, *J* = 10.4 Hz, 1.8 Hz, 1H), 3.60–3.66 (m, 2H), 3.75 (t, *J* = 10.3 Hz, 1H), 3.79 (dd, *J* = 6.3 Hz, 3.0 Hz, 1H), 3.80–3.87 (m, 2H), 3.84 (s, 3H). ^13^C NMR (150 MHz, CD_3_OD): δ 22.65, 26.62, 27.48, 29.85, 30.55, 41.77, 52.41, 53.33, 53.87, 64.68, 65.03, 68.55, 70.20, 72.56, 74.93, 100.20, 171.23, 175.26. ESI-MS m/z calculated for C_21_H_22_O_6_ [M + Na]^+^ 393.1; found 393.1. HRMS (ESI-TOF) *m/z*: [M + H]^+^ Calcd for C_18_H_32_N_4_O_9_ 449.2242; Found 449.2255.

*sym*-Penta-2-[1-[2-((*N*-acetyl-1-methyl-α-neuraminosyl)oxy)ethyl]-1H-1,2,3-triazol-4-yl]ethylcorannulene (**9**). ^1^H NMR (600 MHz, CD_3_OD): δ 1.68 (dd, *J* = 12.8 Hz, 11.6 Hz, 5H), 1.97 (s, 15H), 2.58 (dd, *J* = 12.7 Hz, 3.8 Hz, 5H), 3.07–3.27 (m, 10H), 3.44–3.53 (m, 15H), 3.55–3.68 (m, 15H), 3.60 (s, 15H), 3.73–3.87 (m, 20H), 4.10–4.18 (m, 5H), 4.45–4.53 (m, 10H), 7.63 (s, 5H), 7.79 (s, 5H). ^13^C NMR (150 MHz, CD_3_OD): δ 22.76, 29.15, 34.15, 41.42, 51.41, 53.47, 53.72, 63.89, 64.80, 68.33, 70.13, 72.26, 75.03, 100.24, 124.12, 124.70, 124.72, 130.81, 135.91, 141.29, 170.44, 175.13. HRMS (ESI-TOF) *m/z*: [M + 2H]^+^ Calcd. for C_110_H_150_N_20_O_45_ 1236.5105; found 1236.5132.

*sym*-Penta-2-[1-[4-((*N*-acetyl-1-methyl-α-neuraminosyl)oxy)butyl]-1H-1,2,3-triazol-4-yl]ethylcorannulene (**10**). ^1^H NMR (600 MHz, CD_3_OD): δ 1.25–1.45 (m, 10H), 1.70 (dd, *J* = 12.9 Hz, 11.0 Hz, 5H), 1.78–1.91 (m, 10H), 1.99 (s, 15H), 2.63 (dd, *J* = 12.9 Hz, 4.6 Hz, 5H), 3.22 (appear as t, *J* = 7.3 Hz, 10H), 3.27–333 (overlapped with solvent, 5H), 3.47 (appear as t, *J* = 7.3 Hz, 10H), 3.51 (dd, *J* = 9.1 Hz, 0.9 Hz, 5H), 3.56 (dd, *J* = 10.4 Hz, 1.3 Hz, 5H), 3.60–3.67 (m, 10H), 3.67–3.79 (m, 10H), 3.75 (s, 15H), 3.80–3.85 (m, 10H), 4.25–4.38 (m, 10H), 7.60 (s, 5H), 7.67 (s, 5H). ^13^C NMR (150 MHz, CD_3_OD): δ 22.72, 27.83, 28.11, 29.12, 34.13, 41.67, 50.61, 53.45, 53.84, 64.32, 64.75, 68.49, 70.20, 72.49, 74.93, 100.14, 123.92, 124.33, 130.85, 135.86, 135.86, 141.30, 171.05, 175.21. HRMS (ESI-TOF) *m/z*: [M + 2H]^+^ Calcd. for C_120_H_170_N_20_O_45_ 1306.5887; found 1306.5891.

*sym*-Penta-2-[1-[6-((*N*-acetyl-1-methyl-α-neuraminosyl)oxy)hexyl]-1H-1,2,3-triazol-4-yl]ethylcorannulene (**11**). ^1^H NMR (400 MHz, CD_3_OD): δ 1.09–1.20 (m, 10H), 1.21–1.32 (m, 10H), 1.33–1.43 (m, 10H), 1.66–1.80 (m, 15 H), 2.0 (s, 15H), 2.65 (dd, *J* = 12.8 Hz, 4.6 Hz, 5), 3.16–3.24 (m, 10H), 3.24–3.30 (m, 5H), 3.41–3.50 (m, 10H), 3.51 (dd, *J* = 8.6Hz, 1.8Hz, 5H), 3.54 (dd, *J* = 1.5 Hz, 1.6 Hz, 5H), 3.58–3.76 (m, 20H), 3.79 (s, 15H), 3.81–3.87 (m, 10H), 4.26 (t, *J* = 7.1 Hz, 10H), 7.58 (s, 5H), 7.65 (s, 5H). ^13^C NMR (150 MHz, CD_3_OD): δ 22.66, 26.60, 27.46, 29.83, 30.52, 41.75, 52.39, 53.35, 53.85, 64.66, 65.01, 68.52, 70.17, 72.54, 74.91, 100.17, 171.20, 175.24. HRMS (ESI-TOF) *m/z*: [M + 2H]^+^ Calcd. for C_130_H_190_N_20_O_45_ 1376.6670; found 1376.6679.

*sym*-Penta-2-[1-[2-((*N*-acetyl-α-neuraminosyl)oxy)ethyl]-1H-1,2,3-triazol-4-yl]ethylcorannulene (**12**). ^1^H NMR (600 MHz, D_2_O): δ 1.49 (appear as t, *J* = 12.4 Hz, 5H), 1.99 (s, 15H), 2.58 (dd, *J* = 12.3 Hz, 4.8 Hz, 5H), 3.05–3.25 (m, 10H), 3.30–3.40 (overlapped with solvent, 10H), 3.53 (dd, *J* = 8.7 Hz, 1.7 Hz, 5H), 3.55–3.65 (m, 15H), 3.67–3.63 (m, 10H), 3.74–3.78 (m, 5H), 3.80 (dd, *J* = 11.1 Hz, 2.6 Hz, 5H), 3.93–4.02 (m, 5H), 4.26–4.46 (m, 10H), 7.33 (s, 5H), 7.54 (s, 5H). ^13^C NMR (150 MHz, CD_3_OD): δ 50.19, 51.78, 62.50, 62.59, 68.08, 68.12, 71.57, 72.59, 100.45, 122.86, 124.11, 129.06, 133.42, 139.67, 147.20, 173.21, 174.92. HRMS (ESI-TOF) *m/z*: [M + 2H]^+^ Calcd for C_105_H_140_N_20_O_45_ 1201.4714; Found 1201.4736.

*sym*-Penta-2-[1-[4-((N-acetyl-α-neuraminosyl)oxy)butyl]-1H-1,2,3-triazol-4-yl]ethylcorannulene (**13**). ^1^H NMR (600 MHz, CD_3_OD): δ 0.77–1.04 (m, 10H), 1.41–1.66 (m, 10H), 1.53 (appear as t, *J* = 12.2 Hz, 5H), 2.02 (s, 15H), 2.65 (dd, *J* = 12.3 Hz, 4.7 Hz, 5H), 3.09–3.29 (m, 15H), 3.29–3.48 (m, 15H), 3.53–3.68 (m, 20H), 3.68–3.87 (m, 5H), 3.73 (appear as t, *J* = 10.1 Hz, 5H), 3.81 (dd, *J* = 11.8 Hz, 2.2 Hz, 5H), 3.98–4.29 (m, 10H), 7.29 (s, 5H), 7.41 (s, 5H). ^13^C NMR (150 MHz, CD_3_OD): δ 22.00, 25.31, 26.04, 27.12, 31.99, 40.26, 49.46, 51.90, 62.45, 63.35, 68.09, 68.28, 71.65, 72.53, 100.47, 123.29, 123.53, 129.43, 133.53, 139.80, 146.91, 173.47, 175.03. HRMS (ESI-TOF) *m/z*: [M + 2H]^+^ Calcd for C_115_H_160_N_20_O_45_ 1271.5496; found 1271,5494.

*sym*-Penta-2-[1-[6-((*N*-acetyl-1-methyl-α-neuraminosyl)oxy)hexyl]-1H-1,2,3-triazol-4-yl]ethylcorannulene (**14**). ^1^H NMR (600 MHz, CD_3_OD): δ 0.53–0.72 (m, 10H), 0.84–0.98 (m, 10H), 1.03–1.16 (m, 10H), 1.29–1.45 (m, 10H), 1.57 (t, *J* = 12.1 Hz, 5H), 2.02 (s, 15H), 2.69 (dd, *J* = 12.6 Hz, 4.7 Hz, 5H), 3.03–3.17 (m, 10H), 3.17–3.30 (m, 15H), 3.45–3.52 (m, 5H), 3.57 (dd, *J* = 9.1 Hz, 1.9 Hz, 5H), 3.59–3.68 (m, 15H), 3.75 (appear as t, *J* = 10.1 Hz, 5H), 3.78–3.86 (m, 10H), 4.0–4.16 (m, 10H), 7.17 (s, 5H), 7.23 (s, 5H). ^13^C NMR (150 MHz, CD_3_OD): δ 22.03, 24.33, 24.93, 26.95, 28.62, 29.15, 31.83, 40.53, 49.78, 51.99, 62.48, 64.52, 68.16, 68.32, 71.74, 72.54, 100.59, 122.72, 123.29, 128.85, 133.17, 139.25, 146.83, 173.47, 175.04. HRMS (ESI-TOF) *m/z*: [M + Na]^+^ Calcd for C_125_H_180_N_20_O_45_ 1341.6279; found 1341.6302.

## Figures and Tables

**Table 1:** Data collection and refinements statistics for CVA24v in complex with **14**. Values in parentheses are for the highest resolution shell.

	CVA24v:14 Complex
Resolution (Å) 50	50–1.78 (1.89–1.78)
Space group	I222
Unit cell	
a, b, c (Å)	305.13, 365.55, 366.71
No of reflections	14,772,705 (2,349,016)
unique reflections	1,921,680 (308,924)
Redundancy	7.7 (7.6)
Completeness (%)	99.9 (99.7)
I/σ (I)	6.6 (1.1)
CC1/2	98.6 (50.6)
Wilson B (Å^2^)	21.9
Rmeas (%)	28.2 (142.8)
Rwork (%)	15.8
Rmsd	
Bond angle (°)	1.36
Bond length (Å)	0.005
Average B-Factor (Å^2^)	
CVA24v (VP1/VP2/VP3/VP4)	35/33/32/40
ligand	55
water	45
Ramachandran	
Favoured (%)	94.0
Outlier (%)	1.0

**Table 2:** Cryo-EM data collection and image processing information for CVA24v in complex with compound **14**.

Hardware	
Microscope	Titan Krios
Detector (mode)	K2 super resolution
Accelerating voltage (ke^−^V)	300
Pixel size (Å)	0.82
Data acquisition parameters	
Nominal magnification	165 k
Spot size	7
Illuminated area	1.31
Dose	
Square pixel (Å^2^)	0.67
Dose per physical pixel per second	3.28
Dose per Å^2^/s	4.89
Exposure time (seconds)	10
Total dose (e/Å^2^)	48.9
Number of fractions	50
Dose per fraction (e/Å^2^)	0.97
EPU parameters	
Defocus range (−μm)	−0.5 to −2.3 (0.2 increments)
Autofocus	After distance, 10 μm
Drift measurement	Each grid square, 0.05 nm/s
Delay after stage shift	10 s
Delay after image shift	na
Exposures per hole	na
Apertures (size in microns)	
C1	2000
C2	70
C3	2000
Objective	100
Image processing	
Movies (No.)	1443
Initial particle images (No.)	23,952
Final particle images (No.)	8865
Map resolution (Å)	2.3
FSC threshold	0.143
Map sharpening B-factor (Å^2^)	−49
Symmetry imposed	I1

## Data Availability

Data are contained within the article. The EM density map for CV-A24v in complex with compound **14** has been deposited to the Electron Microscopy Data Bank under the accession codes EMD-13025, and to the Protein Data Bank under the accession code 7OJ7.

## Supplementary Materials

The following are available online at https://www.mdpi.com/article/10.3390/ijms22168418/s1.
