# Supplementary material for: Exploring the Effect of Structure-Based Scaffold Hopping on the Inhibition of Coxsackievirus A24v Transduction by Pentavalent N-Acetylneuraminic Acid Conjugates
Source: Int J Mol Sci. 2021 Aug 5;22(16):8418. doi: 10.3390/ijms22168418 (PMC8395083; doi:10.3390/ijms22168418)
Supplement: Supplementary file 1 [file ijms-22-08418-s001.zip › ijms-1269426-supplementary.pdf]

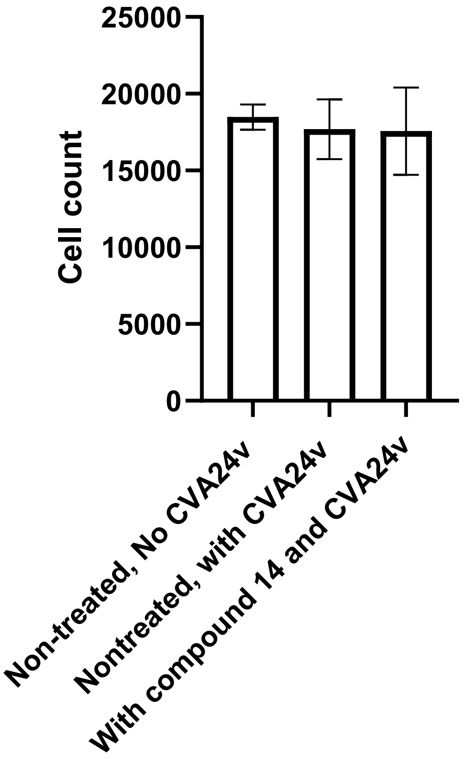

Figure S1. ME1071 is not toxic towards human corneal epithelial cells based on cell counting. Cell viability was studied by counting Hoechst stained cells from the highest concentration of compound 14 (1.25 mM) compared to non-treated cells and non-treated cells with only CVA24v infection. The experiment was performed once in duplicate.

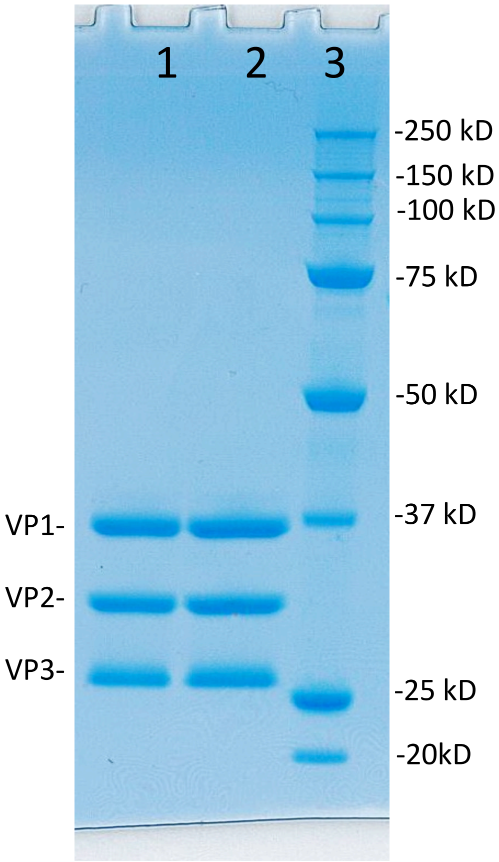

Figure S2. Coomassie stained protein gel indicates high level of CVA24v purity. After ultracentrifugation, purified and concentrated CVA24v samples were loaded on a 10% bis-tris gel (Invitrogen) and ran on at 200 V in 3-(N-morpholino)propanesulfonic acid (MOPS) buffer (Invitrogen). After 50 min, the gel was stained with Pageblue protein stain solution (Thermo Fisher) for 24 hours before destaining with water. Lane 1 and 2 show the capsid proteins VP1, VP2 and VP3 (CVA24v from several purifications, pooled into one sample); Lane 3, protein ladder (Thermo Fisher).
